# Intracavity incoherent supercontinuum dynamics and rogue waves in a broadband dissipative soliton laser

**DOI:** 10.1038/s41467-021-25861-4

**Published:** 2021-09-22

**Authors:** Fanchao Meng, Coraline Lapre, Cyril Billet, Thibaut Sylvestre, Jean-Marc Merolla, Christophe Finot, Sergei K. Turitsyn, Goëry Genty, John M. Dudley

**Affiliations:** 1 Institut FEMTO-ST, Université Bourgogne Franche-Comté CNRS UMR 6174, 25000 Besançon, France; 2 Laboratoire Interdisciplinaire Carnot de Bourgogne, Université Bourgogne Franche-Comté CNRS UMR 6303, 21078 Dijon, France; 3 Aston Institute of Photonic Technologies, Aston University, Birmingham, UK; 4 Aston-NSU International Centre for Photonics, Novosibirsk State University, Novosibirsk, Russia; 5 Photonics Laboratory, Tampere University, FI-33104 Tampere, Finland

**Keywords:** Nonlinear optics, Supercontinuum generation, Solitons

## Abstract

Understanding dynamical complexity is one of the most important challenges in science. Significant progress has recently been made in optics through the study of dissipative soliton laser systems, where dynamics are governed by a complex balance between nonlinearity, dispersion, and energy exchange. A particularly complex regime of such systems is associated with noise-like pulse multiscale instabilities, where sub-picosecond pulses with random characteristics evolve chaotically underneath a much longer envelope. However, although observed for decades in experiments, the physics of this regime remains poorly understood, especially for highly-nonlinear cavities generating broadband spectra. Here, we address this question directly with a combined numerical and experimental study that reveals the physical origin of instability as nonlinear soliton dynamics and supercontinuum turbulence. Real-time characterisation reveals intracavity extreme events satisfying statistical rogue wave criteria, and both real-time and time-averaged measurements are in quantitative agreement with modelling.

## Abstract (web summary)

In this work the authors perform a combined numerical and experimental study of noise-like pulse multiscale instabilities in dissipative laser systems. They reveal an underlying multiscale dynamics and report the observation of rogue wave events.

## Introduction

Recent years have seen tremendous advances in understanding nonlinear complexity through studies in optical systems that allow real-time observation of ultrafast instabilities. For example, studies in optical fibres have yielded new insights into the modulation instability of the nonlinear Schrödinger equation (NLSE)^1–5^, improving our understanding of noise-driven nonlinear dynamics, and stimulating new approaches to classify localised soliton structures using machine learning^6^.

Other studies have focused on instabilities in dissipative soliton lasers, where the laser operation is governed by the balance between nonlinearity and dispersion, and energy input and loss^7–12^. Such lasers are usually configured to produce highly regular pulse trains of ultrafast solitons^13,14^, but they can also exhibit a range of more complex temporal and spectral characteristics. Specifically, the coupling between nonlinearity and dispersion in the cavity can result in instabilities arising from the intrinsic chaotic dynamics of NLSE and NLSE-like systems^15,16^. The particular dynamics that are observed depend on the dimensionality of the system under study^17^, and for certain laser designs, it has been possible to see clear signatures of low-dimensional nonlinear dynamics such as the development of complex temporal pattern formation^18^, and bifurcation routes to chaos^19^.

More commonly, however, fibre lasers possess a very large number of degrees of freedom so that instabilities are high-dimensional such that the particular operating point of stable pulse train generation can be viewed as an attractor in a multi-dimensional parameter space. In some cases, variation of the cavity parameters about these stable points can induce transition into unstable regimes involving interactions between a small number of circulating pulses (typically 1–10) in the cavity. Studying these instabilities with real-time measurement techniques has led to improved insight into processes such as soliton molecule coupling^20–22^, complex temporal pattern formation in lasers^23–25^, soliton explosion and rogue wave emergence^24,26^, and complex intermittence^27,28^. In fact, from a more general perspective, these regimes are neither pure mode-locked pulses, nor continuous-wave generation. These highly complex lasing regimes are characterised by the co-existence of both localised nonlinear structures and linear dispersive waves.

In addition to regimes involving only a small number of interacting pulses, a more complex multiscale laser instability has been shown to involve a much greater number (100–1000) of ultrafast pulses evolving randomly underneath a much broader envelope. This multiscale regime of noise-like pulse operation was first discovered by Silberberg et al. in 1997^29^, and has since been seen in a wide range of different laser configurations, and with both normal and anomalous dispersion^30^. The majority of studies have typically focused on instabilities generating spectral bandwidths of 10s of nm^31–37^, but in a highly nonlinear regime with a significant spectral broadening in the cavity, bandwidths much greater than the gain bandwidth (100s of nm) have been observed^38–41^. In addition to their clear interest from a dynamical systems perspective, such lasers have found several important applications^30,42^. Significantly, while some applications such as tomography and metrology explicitly build on the low temporal coherence of such sources^43^, applications in material processing exploiting the burst-like nature of the pulsed output have also been demonstrated^44^.

Somewhat paradoxically, the large number of experimental studies of noise-like pulse lasers under so many different conditions has made it difficult to clearly identify the underlying physics. Also, the multiscale nature of the laser operation has not always been appreciated in experiments measuring only the envelope or burst output characteristics. Nonetheless, the underlying role of NLSE-like instabilities has been suggested from numerical studies, using both cubic–quintic Ginzburg–Landau equation modelling^45–47^ and iterative cavity simulations^30,31^. Further modelling^48,49^ has revealed how these dynamics can lead to rogue wave statistics, confirming previous experiments in the narrowband noise-like pulse regime^34,35,50^.

Significantly, the results of these previous studies have now extended traditional notions of laser operation beyond average dynamical models to include concepts such as strong dissipation, regenerative saturable absorption^51^, random lasing^52^, and intracavity turbulence^53^. The study of turbulent behaviour in lasers is a subject of particular interest, and can be physically interpreted as a consequence of the large number (~10^4^–10^6^) of interacting frequency modes underneath the evolving broadband field^54–56^. Moreover, linking irregular dynamics to turbulence is also interesting from the perspective of understanding intracavity extreme events, which have already been observed in single-pass experiments^57,58^.

A particular challenge is to understand the dynamics of noise-like pulse lasers generating the broadest bandwidths, because the presence of highly nonlinear fibre in the cavities used suggests a major role played by supercontinuum broadening. In this case, any modelling in a laser context is computationally very expensive, and measuring these multiscale instabilities in the laboratory is also extremely challenging. These different factors clearly represent a serious limitation when a full understanding of such a rich dynamical laser system is of evident interest from both fundamental and applied perspectives.

Here, we report a combined numerical and experimental study of an extreme dissipative soliton noise-like pulse laser generating an output supercontinuum spectrum of ~1000 nm bandwidth, and with intracavity spectral width varying two orders of magnitude over one roundtrip. Our stochastic numerical simulations allow us to identify the origin of the laser instability as due to the sensitivity to noise of nonlinear soliton dynamics, particularly multiple interactions and collisions between incoherently evolving sub-picosecond Raman solitons. Our experiments use time and frequency-domain techniques to characterise the multiscale dynamics, and our simulations reproduce quantitatively the supercontinuum broadening, and the probability distributions of temporal and spectral fluctuations, including rogue wave events.

## Results

### Modelling and dynamics

**Fig. 1:**
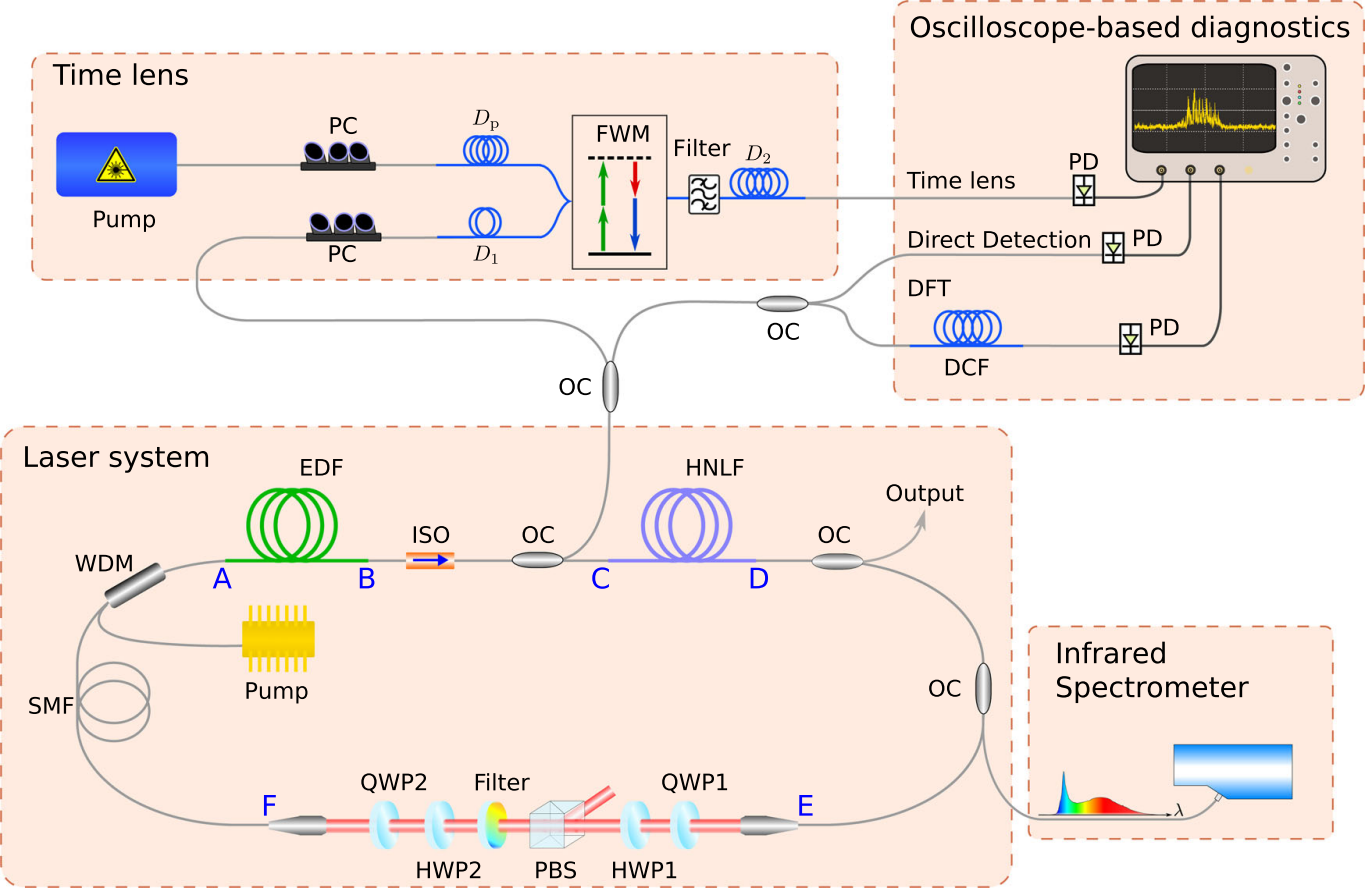
Schematic of the laser system and of the various diagnostics used. Text labels A–F shown on the cavity refer to the different propagation segments as discussed in the text. PC polarisation controller, *D*_*p*_, *D*_1_, *D*_2_ time-lens dispersive elements (see ‘Methods’ section), FWM four-wave mixing, DFT dispersive Fourier transform, DCF dispersion compensating fibre, ISO isolator, HWP half-wave plate, QWP quarter-wave plate, PBS polarising beamsplitter, EDF Erbium-doped fibre, SMF single-mode fibre, HNLF highly nonlinear fibre, WDM wavelength-division multiplexer. OC optical coupler, PD photodiode.

We first use numerical modelling to illustrate the general features of the noise-like pulse instability regime. Figure 1 shows the dissipative laser system upon which our modelling is based. Typical of dissipative soliton systems, we consider a unidirectional ring cavity consisting of a number of discrete segments where the intracavity field experiences qualitatively different evolution^9,13^. The modelling uses an iterative map approach describing each cavity element by a suitable transfer function. For a given set of system parameters, the model seeks convergence to a particular operating state after injection of an initial seed (see ‘Methods’ section). Although scalar models have been shown to reproduce aspects of dissipative soliton laser dynamics qualitatively^59^, for quantitative comparison with experiments we implement a more complete approach based on coupled generalised vector nonlinear Schrödinger equations (NLSE). This was found essential to obtain quantitative agreement with experiments. Indeed, it is important to stress that although mean-field models such as those based on the Ginzburg–Landau equation are able to reproduce general features of dissipative soliton lasers^46^, the iterative cavity approach is necessary when describing a system with such dramatic variation in evolution between the different cavity segments.

The simulations use a standard approach, with initiation from a low amplitude noise seed^60^ injected at point A before the EDF. The intracavity field then evolves over multiple roundtrips until convergence to a particular operating state. For a stable state, the spectral and temporal field characteristics at any point in the cavity reproduce themselves after one roundtrip, whilst in a noise-like pulse state, the temporal and spectral characteristics at any point fluctuate significantly with roundtrip but the energy nonetheless has a well-defined mean. Typical energy fluctuations after the build-up to the noise-like pulse regime are ~10%, and the physical origin of this behaviour is the chaotic nature of the NLSE and gain/loss dynamics when seeded by noise.

In our experiments, segment AB consists of 11 m of Erbium-doped fibre (EDF), segment BC consists of 2.87 m of standard single-mode fibre (SMF), segment CD consists of 10.3 m of highly nonlinear fibre (HNLF), and segments DE and FA consist of 4.45 m and 7.80 m of SMF. A 28.1 cm bulk-optics free space segment EF includes a nonlinear-polarisation rotation-based saturable absorber (using waveplates and a polarisation beamsplitter)^61^, and a narrowband spectral filter to control the bandwidth of the pulses reinjected into the EDF^13,62^. The cavity uses non-polarisation-preserving fibre, and the repetition rate is 5.59 MHz (roundtrip time of ~179 ns.) The dispersion and nonlinearity parameters of the fibres used are given in the ‘Methods’ section. The loss due to splicing and output coupling was ~6 dB (with primary output coupling of 40% in segment DE), and the total energy loss at the spectral filtering stage is ~10 dB (associated with the bandwidth reduction from the supercontinuum spectrum at the HNLF output to the much narrower bandwidth at the EDF input).

Our study of this particular design is motivated by the need to understand noise-like pulse dynamics in the broadband regime. Specifically, the majority of previous studies of noise-like pulse lasers have focussed on narrowband systems with 10s of nm bandwidth^30^, where modelling and simulations have shown how the dynamics arise from the interaction between self-phase modulation and group-velocity dispersion in the cavity^13,30,45–47^, with only minor contributions from higher-order effects. With the addition of anomalous dispersion HNLF, however, the dynamics change qualitatively and quantitatively with the processes of incoherent soliton fission and the Raman soliton self-frequency shift combining to dominate the intracavity spectral broadening^63,64^. Moreover, in contrast to other dissipative soliton laser designs that can exhibit both stable soliton and noise-like pulse operation^65^, the use of such a long length of HNLF results in this system operating only in the noise-like instability regime, irrespective of the laser gain or waveplate orientations. In the simulation results that follow, we therefore focus on the dynamics of this unstable broadband operating state with parameters corresponding to our experiments, although we refer when appropriate to Supplementary Information which shows simulations for other parameters in order to clarify certain features of the underlying physics.

**Fig. 2:**
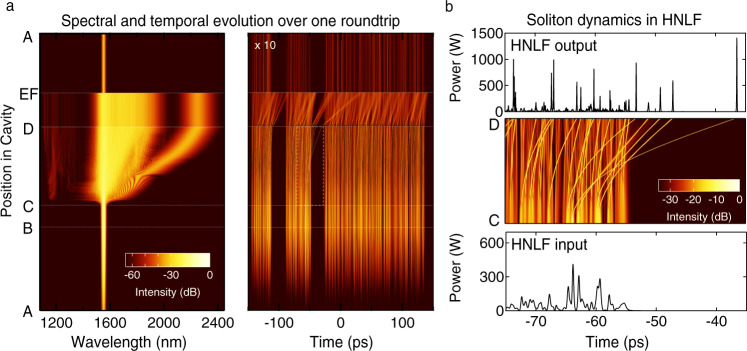
Typical results from numerical simulations showing the evolution over one roundtrip in the noise-like pulse regime. **a** False-colour evolution plots in (left) spectral and (right) temporal domains to illustrate the dramatic differences in evolution in the different cavity segments. The labels A–F refer to the cavity schematic in Fig. 1. Because of the significant loss due to the spectral filter in the bulk segment, the temporal intensity in segment FA is scaled by a factor of 10×. **b** The HNLF evolution in segment C, D over an expanded 40 ps time window to clearly reveal the soliton dynamics and Raman soliton evolution. This is the region indicated by the dashed white lines in **a**. This figure also shows the intensity profiles at the input and output of the HNLF.

We begin by showing typical simulation results in the noise-like pulse regime for our experimental parameters as above. Figure 2a shows typical simulated spectral and temporal evolution over one cavity roundtrip, after build-up when the pulse has entered the regime of constant mean energy. These results were obtained after scanning the simulation parameters to obtain energy and noisy envelope durations comparable to the experiment (see ‘Methods’ section), and correspond to a mean intracavity energy (over 1000 roundtrips) at the EDF output of 10.5 nJ. We plot the total intensity of both polarisation components (see ‘Methods’ section). The different propagation steps A–F refer to the different points in the cavity shown in Fig. 1.

**Fig. 3:**
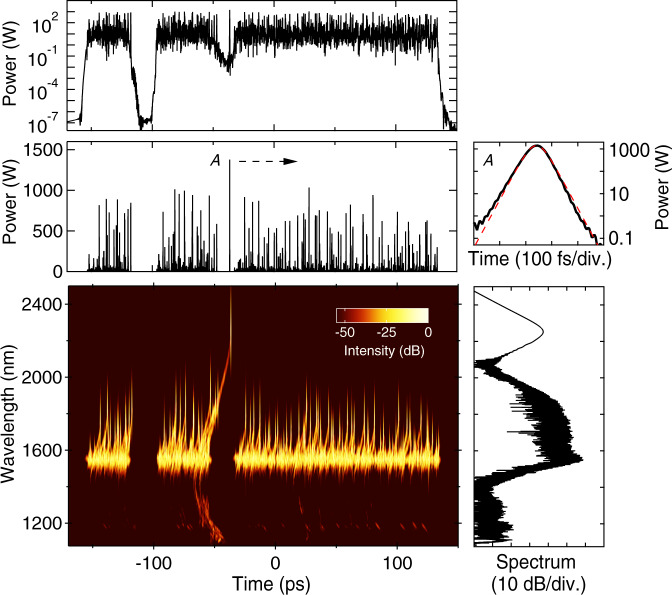
Simulated single-shot spectrogram at the HNLF output. The spectrogram representation is shown together with the projected temporal intensity (top, shown on both linear and logarithmic scales) and spectrum (right). The highest temporal intensity peak from simulation (*A*) is shown in an expanded view on a logarithmic scale (black solid line) and compared with a hyperbolic secant soliton fit (red dashed line). The Supplementary Movie shows the spectrogram evolution over one roundtrip.

**Fig. 4:**
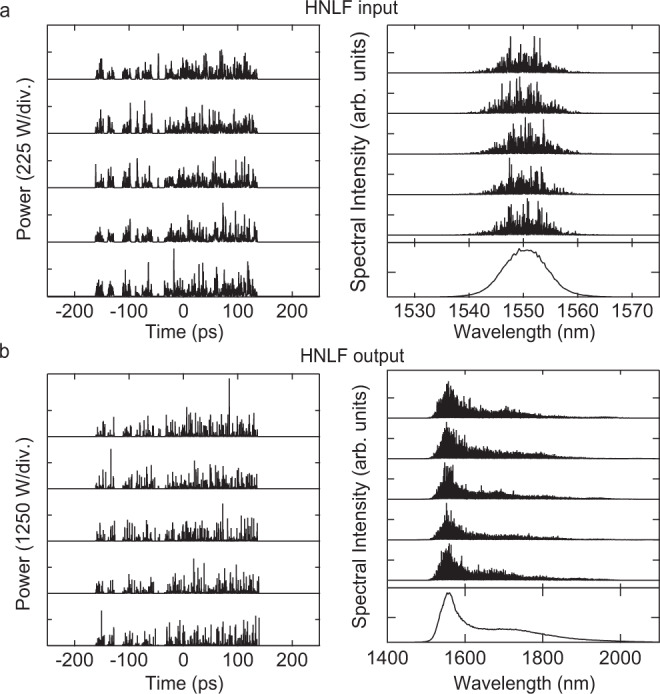
Simulated temporal and spectral characteristics showing shot-to-shot fluctuations. Results from numerical simulation plotting temporal and spectral characteristics over 5 roundtrips at **a** the HNLF input and **b** the HNLF output. For the spectral results, we also plot the mean spectrum computed over 1000 roundtrips (bottom subfigure). Because we plot results on a linear scale here to highlight the shot-to-shot fluctuations, we do not include the wavelength range of the low amplitude dispersive wave components.

In the frequency domain, Fig. 2a shows how the spectral characteristics vary significantly over one roundtrip. We see amplification in the EDF (segment AB), dramatic supercontinuum spectral broadening in the HNLF (segment CD), and the strong effect of spectral filtering in the bulk segment EF. Indeed the spectral extent varies by two orders of magnitude over one roundtrip, from ~10 nm FWHM immediately after the spectral filter, to a supercontinuum with spectral components spanning ~1000 nm at the HNLF output. In segment DE, the reduced nonlinearity of the SMF (coupled with reduced power due to output coupling) yields essentially linear propagation without additional spectral broadening. As we will see in Figs. 3 and 4, the shot-to-shot spectra exhibit significant fine structure, but this is not apparent in Fig. 2 because of the false-colour visualisation used.

The associated time-domain evolution in Fig. 2a reveals significant variation in the sub-picosecond temporal structure over one roundtrip, while the slower ~300 ps envelope remains largely unchanged. The simulations also reveal the presence of clusters of solitons under the envelope (see for example timebase values in the range ~[−150, −50] ps), an effect previously suggested by low-resolution temporal measurements, and associated with an additional scale of time-domain structure^66^. Note that we also confirm the presence of these clusters in our experiments below.

After the HNLF propagation phase, the linear evolution in the subsequent SMF segment DE is associated with dispersive broadening of the solitons formed in the HNLF. Note that the apparent temporal refraction of the pulse trajectories across the HNLF-SMF boundary (point D) arises from the differences in group-velocity dispersion in the two fibres. We also see how the spectral filtering in the bulk segment EF has a significant effect on the temporal evolution by removing all field components outside the filter bandwidth, particularly the long-wavelength shifted highest intensity Raman solitons. To more clearly show the temporal evolution after this filtering step, the intensity colourmap in segment FA has been scaled by a factor of 10×.

Figure 2b provides an expanded view of the evolution in the HNLF segment CD over a 40 ps time window, together with the input and output intensity profiles. These results clearly reveal the dominant physics associated with the temporal compression of the *T*_FWHM_ ~ 500 fs random pulses at the input to yield ejection of strongly localised sub-100 fs pulses that then undergo typical supercontinuum dynamics of soliton collisions and Raman shifting to longer wavelengths^67^. Indeed, using *P*_0_ ~ 70 W and *T*_0_ = *T*_FWHM_/1.76 ~ 300 fs as estimates of the mean peak power and duration of the random pulses at the input to the HNLF, the corresponding soliton number is $N={(\gamma {P}_{0}{T}_{0}^{2}/| {\beta }_{2}| )}^{1/2}\approx 4.5$, supporting this interpretation^68^. Associated with these multiple soliton dynamics in the HNLF is the generation of multiple dispersive waves at shorter wavelengths extending to below ~1100 nm, as seen clearly in the spectral evolution in Fig. 2a. We note here that the evolution of the Raman soliton trajectory in Fig. 2a appears to show spectral broadening, but this is in fact an artefact associated with the fact that we are plotting spectral evolution against wavelength. When plotted against frequency, the soliton bandwidth remains constant once it has clearly separated from the central spectral region. On the other hand, soliton evolution in the presence of the Raman effect and higher-order dispersion can exhibit complex accelerating beam characteristics^69^, and this is especially apparent in the trajectories of the time-domain solitons seen in the false-colour plot in Fig. 2b.

The results in Fig. 2b clearly show that the input field to the HNLF consists of a large number of irregular localised pulses. As a result, the field injected in the HNLF does not excite narrowband processes such as modulation instability^11,70,71^, but rather the dynamics are dominated by incoherent soliton fission. In this case, the decoherence that develops during propagation arises from the effect of noise on soliton interaction and collisions^63,64^ and these results highlight the importance of incoherent soliton fission in noise-like pulse lasers. Of course, under certain conditions, both modulation instability and incoherent soliton fission yield similar characteristics with very large numbers of interacting solitons, and in this case, a description in terms of soliton turbulence is more appropriate in both cases^54–56,70,72,73^. This is also the regime where extreme event rogue wave statistics can be observed^57,58,74,75^.

Additional insight into these dynamics is shown in Fig. 3. Here we plot the computed time-frequency spectrogram at the HNLF output, together with the corresponding temporal and spectral intensity profiles (see ‘Methods’ section). Note that we also display the temporal intensity on a logarithmic axis to illustrate the strongly localised burst-like nature of the noise-like pulse emission. The spectrogram reveals a time-frequency structure typical of Raman-dominated supercontinuum dynamics^63^, highlighting how each of the solitons generates its own dispersive wave component, and also highlighting the prominent dispersive wave tail associated with the longest wavelength soliton. An accompanying animation (Supplementary Movie 1) shows the evolution of the spectrogram over one cavity roundtrip, and is valuable in revealing the specific dynamics occurring in each segment. To confirm the soliton nature of the sub-picosecond peaks in the temporal intensity, the highest peak (*A*) is shown in expanded view on a logarithmic scale (black solid line), and compared with a hyperbolic secant soliton fit (red dashed line). In fact, since the spectrogram allows us to determine the wavelength associated with each temporal peak, we can readily compute the associated soliton number using wavelength-corrected HNLF nonlinearity and dispersion parameters (see ‘Methods’ section). This analysis yields *N* ~ 1 for all the strongly localised temporal peaks seen at the HNLF output, further confirming the importance of intracavity soliton dynamics.

The Raman self-frequency shift of the ejected solitons yields the long-wavelength spectral extension. And indeed, reaching short wavelengths around 1100 nm would not be possible without the associated dispersive wave generation. However, the presence of Raman scattering is not in itself a necessary condition to observe noise-like pulse operation, and simulations with no HNLF in the cavity still show unstable pulse characteristics for certain parameter regimes. However, without the HNLF, the system operates only in the narrowband regime (see Supplementary Information and Supplementary Fig. 1), as seen in a number of previous studies^29,31–37^. The essential design parameter here is of course the HNLF length, but additional simulations show that broadband noise-like pulses can be generated with short lengths of HNLF down to ~1 m (Supplementary Fig. 2). Of course, the role of the Raman soliton dynamics and the overall bandwidth increase with longer lengths of HNLF, as would be expected on physical grounds (Supplementary Fig. 3).

The key characteristic of the noise-like pulse regime is of course the dramatic shot-to-shot fluctuations, and to illustrate random field evolution over sequential roundtrips, Fig. 4a, b plot the temporal and spectral characteristics at the input and output of the HNLF respectively for 5 sequential roundtrips (after converging to constant mean energy.) The parameters here are the same as in Figs. 2 and 3 corresponding to the 10.3 m HNLF used in the experiment. These results clearly illustrate the dramatic shot-to-shot variation associated with this operating regime, and the temporal profiles, in particular, show the expected characteristics of the noise-like pulse regime. Specifically, the temporal profiles (shown over a 500 ps span within the 1.3 ns computation window—see ‘Methods’ section) clearly show the large number (~300) of chaotic soliton peaks whose intensities vary dramatically from shot-to-shot. The spectral characteristics also show significant fluctuating fine structure. Note that for the spectral results, we also plot the mean spectrum computed over a much larger number of roundtrips (1000) to highlight the fact that these fluctuations are not at all apparent using average spectral measurements. Comparing Fig. 4a, b further highlights the extent of the HNLF spectral broadening.

### Experiments and comparison with simulations

Both time-averaged and statistical predictions of our numerical model have been confirmed using experiments. The overall design of our laser system has been described above (and in Fig. 1). The EDF was pumped at 976 nm, with a pump power threshold of 40 mW for pulsed operation, although we did not observe stable mode-locked operation at any pump power or waveplate orientation^28^. On the other hand, over a wide range of pump powers up to 500 mW, suitable adjustment of the waveplates yielded noise-like pulse operation with chaotic envelope emission at the cavity repetition rate, and broadband spectral output. The results reported below at a pump power of 225 mW are typical of this regime. For this value of pump, the average power at the primary output in branch DE was 13 mW (see ‘Methods’ section).

We used a range of diagnostics to characterise the laser output. The real-time shot-to-shot characterisation was possible for the ~10 nm (3 dB) bandwidth pulses input to the HNLF (point C in Fig. 1) using several different techniques: direct measurement of overall envelope fluctuations via a fast photodiode, a time-lens system to measure sub-picosecond soliton structure on the circulating pulses, and a dispersive Fourier transform (DFT) setup for spectral measurements. At the HNLF output, the large spectral bandwidth of the supercontinuum pulses exceeded the measurement bandwidths of our real-time devices, but time-averaged measurements of the spectra over the full bandwidth of 1000–2100 nm were performed using an OSA and a near-infra-red spectrometer. In this context, we note that temporal measurements using intensity autocorrelation are of partial utility, as they indicate just the presence of instability through a coherence spike on a broad envelope, and give only an indirect measure of the average sub-structure pulse duration^30^.

**Fig. 5:**
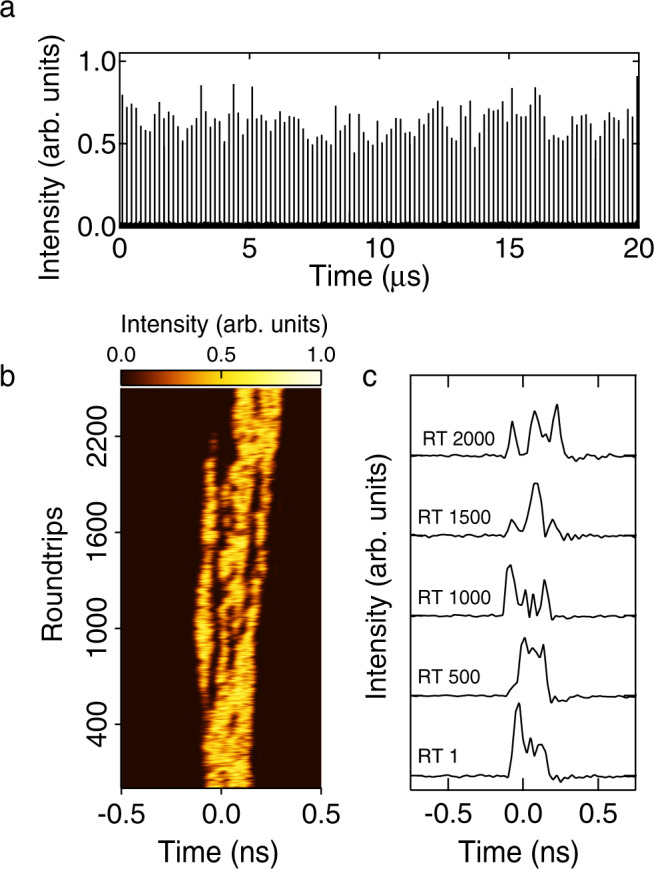
Photodiode characterisation results after the EDF and before injection in the HNLF. **a** Measured pulse train plotted over a 20 μs time span. **b** False-colour representation of the temporal evolution of the pulse envelope over 2500 roundtrips. **c** Temporal intensity profiles at roundtrip (RT) 1, 500, 1000, 1500 and 2000.

Figure 5 shows the instability properties of the laser measured using direct photodetection. Figure 5a plots data from a fast photodiode over a time span of 20 μs to illustrate the emission of highly unstable pulses at the 5.59 MHz repetition rate (period of ~179 ns). Although these measurements do not resolve structure faster than the system response time of ~25 ps, we can still accurately characterise the ~300 ps envelope, and, within the detection bandwidth, we can even observe variation in the envelope structure with roundtrip. Figure 5(b) unwraps the raw photodiode data to plot the roundtrip variation of the envelope, and reveals the presence of sub-cluster structure evolving over ~500–1000 roundtrips. Although these results clearly reveal shot-to-shot instability on the envelope, the temporal resolution is limited, as seen in the selection of intensity profiles plotted in Fig. 5c. However, these results are important, because the sub-clusters seen on the envelope confirm previous studies of similar envelope sub-structure^66^, and are a clear illustration of random pattern formation and multiscale dynamics in a dissipative soliton laser^23–25^. In particular, although they are unstable, the sub-clusters are relatively long-lived over multiple roundtrips ~100s of μs and they consist of a large number of localised solitons of ~500 fs duration. Of course, the photodiode measurements are unable to resolve both these long-timescale characteristics as well as to directly measure the ultrafast soliton structure, and it is for this reason that we performed time-lens measurements for shot-to-shot characterisation on the sub-ps scale.

The time-lens system used is described in the ‘Methods’ section and had a temporal resolution of ~340 fs and a physical (i.e. demagnified) measurement window of ~100 ps. Although the finite acquisition window precludes characterisation across the full ~300 ps pulse envelope, by operating the time-lens asynchronously^10^, we are nonetheless able to sample the random pulse structure across the envelope over multiple laser roundtrips. Because of the time-lens measurement bandwidth of ~12 nm, real-time characterisation was possible only for the input pulses to the HNLF, but as we shall see, these measurements clearly reveal both the sub-ps pulse structure and the rogue wave statistics expected from the underlying nonlinear dynamics.

**Fig. 6:**
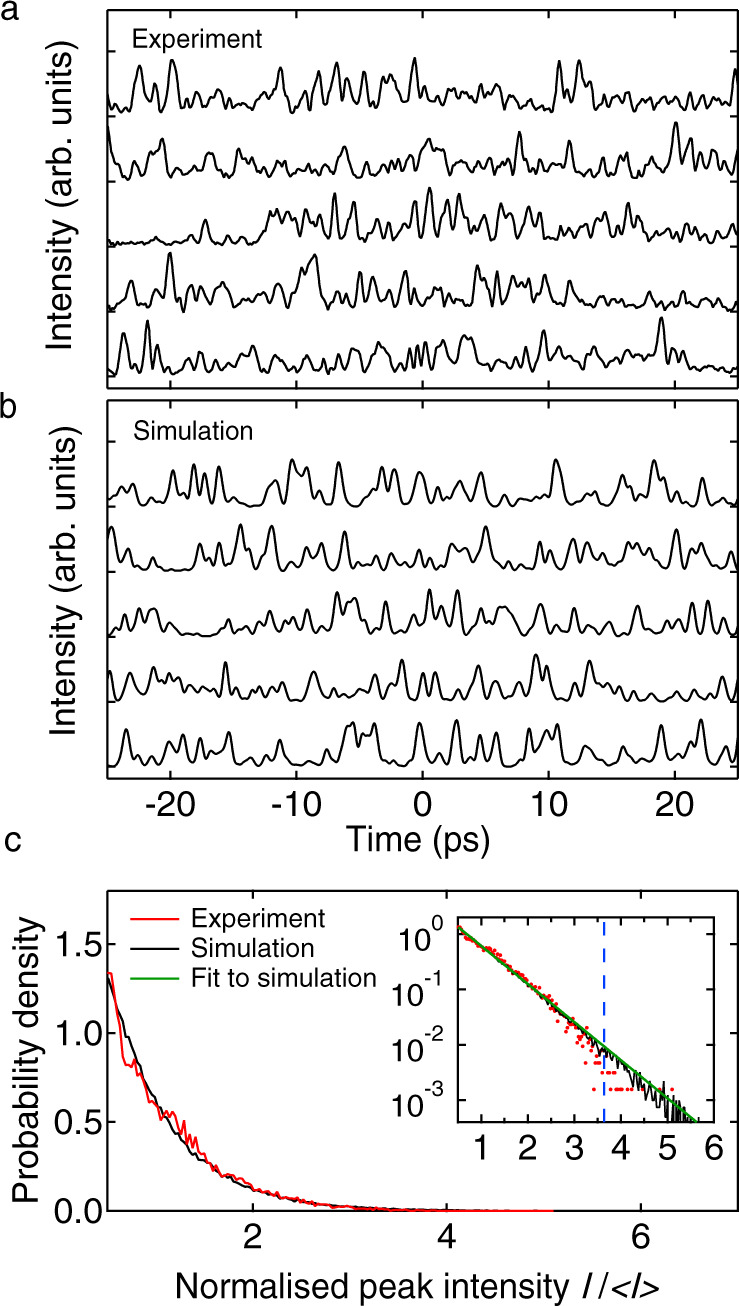
Time lens results from experiment and simulation. Random intensity fluctuations for 5 typical roundtrips at the HNLF input from **a** experiment, and **b** simulation. Experimental data show time-lens results plotted against time after scaling to account for the temporal magnification. **c** Normalised probability density for the peak intensities from the experiment (red line) and simulation (black line). Both experiment and simulation are normalised relative to the mean of the intensity time series. The inset plots the probability density functions on a logarithmic scale to highlight quantitative agreement over 3 orders of magnitude, including the highest intensity peaks. The dashed blue line shows the computed rogue wave threshold from the experiment (which on this graph would coincide with that from simulation). The green line shows a fit to a negative exponential distribution as discussed in the text.

Figure 6a shows 5 typical results from the time-lens measurements, plotting the temporal profiles (after demagnification) over a 50 ps timebase. For comparison, 5 typical results from numerical simulations are also shown in Fig. 6b, and we clearly see the strong visual similarity between the measured and simulated intensity peaks. To compare these results more quantitatively, Fig. 6c plots the computed probability density functions for the peak intensities computed from the experiment (red) and simulation (black). These density functions were computed from a time series of 21,000 temporal peaks measured experimentally, and 135,000 peaks analysed from simulation. The intensity normalisation was relative to the mean of each respective time series. The inset plots the density functions on a logarithmic scale.

We first note excellent agreement between the simulated and experimental results, with simulations reproducing the experimentally measured intensity probability density function over 3 orders of magnitude. The inset also shows a very good agreement between the simulation statistics and a negative exponential fit (green line), where an exponential distribution is expected here on physical grounds given the randomness of the intensity fluctuations in the noise-like pulse regime. Indeed, such negative exponentially distributed statistics have been seen in previous experiments studying statistics of modulation instability and noise-like pulse lasers^6,35^. In addition to clearly revealing the statistical behaviour of the sub-ps soliton structure of noise-like pulses, these results also strongly confirm the fidelity of the model in reproducing the time-domain properties of the laser system.

We also see (from both simulation and experiment) that a significant fraction of the measured intensity peaks exceeds twice the significant intensity threshold (see ‘Methods’ section), thus meeting the statistical criterion to be formally classified as intracavity rogue waves. Indeed, the rogue wave intensity thresholds computed from the experiment (${I}_{{{{{{{{\rm{RW}}}}}}}}}^{\ \exp }=3.64$) and simulation (${I}_{{{{{{{{\rm{RW}}}}}}}}}^{\ {{{{{{{\rm{sim}}}}}}}}}=3.68$) are in very good agreement, and the experimental threshold is shown in the figure as the dashed blue line. From this data, it is also straightforward to calculate the fraction of temporal peaks satisfying the rogue wave criterion, which yields 0.43% from simulation, and 0.10% from the experiment.

When we are in the noise-like pulse regime generating broadband spectra, the temporal probability distribution as shown in Fig. 6c is largely insensitive to small changes in the design parameters. Specifically, performing numerical simulations with small changes (within ± 10%) in parameters such as *f*_R_, *E*_sat_, *g*_0_, and the length of the HNLF have a negligible effect on the temporal pulse characteristics, and thus the negligible effect on the associated probability density function. This is also seen in experiments when we make small changes in pump power for example. We attribute this insensitivity to the fact that our cavity configuration contains a long length of HNLF such that we observe noise-like pulse behaviour at all pump powers above the threshold.

**Fig. 7:**
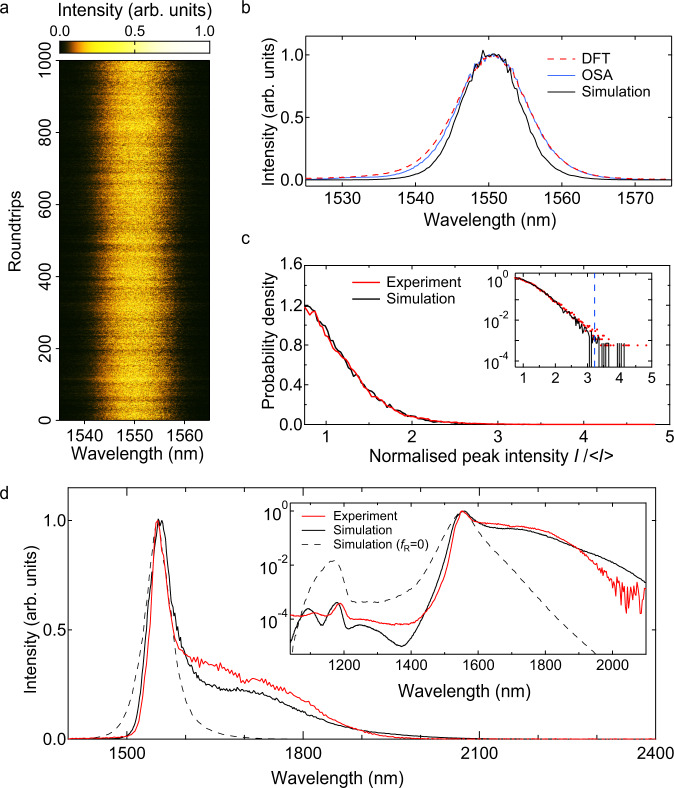
Spectral characterisation and comparison with simulation. **a** False-colour plot of measured shot-to-shot spectral fluctuations over 1000 roundtrips at the HNLF input. **b** Corresponding mean spectrum from DFT (red dashed), time-averaged OSA measurements (blue) and simulations (black). **c** Computed probability density functions for the peak intensities from the experiment (red) and simulation (black). Both experiment and simulation are normalised relative to the mean of the intensity time series. The inset plots the results on a logarithmic scale to highlight quantitative agreement over 3 orders of magnitude, including the highest intensity peaks. The dashed blue line shows the experimental rogue wave threshold which on this graph would coincide with that from simulation. **d** Broadband supercontinuum spectra at the HNLF output, comparing experiment (red) and simulation (black). The dashed line shows simulation results without the Raman term (*f*_R_ = 0). The inset plots the spectra using a logarithmic scale showing also an expanded wavelength range (combining measurements from the Anritsu MS9710B OSA and the NIRQuest512 spectrometer). Simulations reproduce spectral features over a span approaching ~1000 nm.

Complementing the time-domain measurements in Fig. 5, we were also able to record real-time spectral data. Specifically, the DFT method was used to record spectral fluctuations at the HNLF input, and Fig. 7a shows a false-colour representation of the shot-to-shot variation measured over 1000 roundtrips. The fidelity of the DFT method was confirmed by computing the average over this measurement ensemble and comparing with an averaging optical spectrum analyser. The agreement between these measurements is clear from Fig. 7b, and this figure also shows the average input spectrum obtained from simulations. There is a small difference between the experimental and simulation bandwidth, but this does not influence the interpretation of our results.

As we saw in the simulation results in Fig. 4, the shot-to-shot spectra also show a strong sub-structure of spectral peaks. Although analysing spectral peak statistics does not have the same direct physical interpretation in terms of soliton dynamics as analysis of temporal peaks, several studies have characterised spectral peak fluctuations in the context of identifying frequency-domain rogue wave events^34,50^. From our real-time spectral measurements, we computed peak statistics in a similar way as for the temporal data, and the corresponding probability density function is shown in Fig. 7b. Here we also compare with simulation, but note that the simulated spectra were convolved before peak analysis with a response function to model the spectral resolution of the experimental DFT data (see ‘Methods’ section) The figure plots results on both linear and logarithmic scales, and as with the temporal data, there is a very good agreement. The rogue wave spectral peak intensity threshold computed from the experiment was (${S}_{{{{{{{{\rm{RW}}}}}}}}}^{\ \exp }=3.22$) and simulation was (${S}_{{{{{{{{\rm{RW}}}}}}}}}^{\ {{{{{{{\rm{sim}}}}}}}}}=3.20$); the experimental threshold is shown in the figure as the dashed blue line. From this data, it is also straightforward to calculate the fraction of spectral peaks satisfying the rogue wave criterion, which yields 0.04% from simulation, and 0.07% from the experiment. These density functions were computed from a time series of 57,500 spectral peaks measured experimentally, and 44,900 peaks analysed from simulation. The intensity normalisation was relative to the mean of each respective time series.

Finally, we discuss Fig. 7d, which shows the measured average spectrum at the HNLF output (red curve). The inset uses a logarithmic scale, and plots over an extended wavelength range to show the dispersive wave components by combining measurements from the Anritsu MS9710B OSA and the NIRQuest512 spectrometer (see ‘Methods’ section). The spectrum is highly asymmetric, consisting of a primary spectral peak of ~40 nm bandwidth, an extended long-wavelength tail, as well as a short wavelength dispersive wave structure. The overall span of the measured spectrum approaches ~1000 nm.

These experimental results are reproduced in our modelling, where the solid black line shows the simulated average spectrum (computed over 1000 roundtrips) based on the full generalised propagation model. There is very good agreement between experiment and simulation and, given the complexity of the dissipative soliton system that we are modelling, we stress at this point the significance of these results. Specifically, although there have been a large number of distinct experimental and numerical studies of noise-like lasers, our simulations here quantitatively reproduce the observed energy characteristics, the time-averaged broadband spectrum over a span approaching 1000 nm, as well as the intensity statistics of random sub-picosecond soliton structures. Moreover, they clearly show the role of soliton dynamics, allowing us to physically associate the long-wavelength tail with the random evolution dynamics of Raman solitons, and the short wavelength structure from the corresponding dispersive waves. Note that the modulation in the dispersive wave spectral structure arises from cross-phase modulation from the generating soliton pulse^76^, sometimes described as analogous to event horizon dynamics^77^. The importance of the Raman dynamics is explicitly seen by comparing experiment to simulations performed in the absence of the Raman contribution (i.e. *f*_R_ = 0). These are shown as the dashed black line, and we clearly see that the long-wavelength spectral extension, in this case, is absent. Note that the stronger dispersive wave component here arises from more efficient dispersive wave energy transfer when there is no Raman-induced wavelength shifting.

## Discussion

Despite the fact that the incoherent noise-like pulse regime of optical fibre lasers has been observed for nearly 25 years, its physics has been understood in only very general terms. Indeed, the very appellation of noise-like is a very generic description, and does not provide information or even any hints about the pulse dynamics or possible underlying instability mechanism.

The experiments and modelling reported here have addressed this problem for the particular case of a broadband dissipative soliton laser operating in a highly nonlinear regime. Our experiments reveal multiple ultrafast localised structures with random characteristics typical of soliton turbulence, and our simulations reveal the associated intracavity dynamics of soliton fission, Raman evolution and supercontinuum generation. The simulations predict both time-averaged and statistical properties in quantitative agreement with experiments.

From an experimental viewpoint, our results have provided a further example of the great utility of ultrafast real-time measurements in providing new insights into complex nonlinear dynamics in optical fibre systems. Although we have applied real-time characterisation to the particular case of a broadband incoherent dissipative soliton laser, these methods are general, and we expect future experiments to study similar instability mechanisms in regimes of noise-like pulse laser operation with narrower bandwidths. In particular, developing a more complete understanding of the relative importance of modulation instability and incoherent soliton fission in driving irregular cavity dynamics is likely to be an important area of future study.

Perhaps most significantly, our results suggest that for this case of broadband instability in a highly nonlinear regime, we can clarify the noise-like pulse regime as one where intracavity supercontinuum dynamics play a dominant role. This work extends our knowledge of dissipative soliton systems, and further highlights the rich dynamics of laser oscillators when operated far from a weakly-perturbative dynamical regime.

## Methods

### Numerical modelling

Numerical simulations of laser pulse evolution used an iterative map with appropriate transfer functions for each cavity element^78^. We write the pulse amplitude as ${{{{{{{\bf{A}}}}}}}}(z,T)=\hat{{{{{{{{\bf{x}}}}}}}}}\ u(z,T)+\hat{{{{{{{{\bf{y}}}}}}}}}\ v(z,T)$, where *u*(*z*, *T*) and *v*(*z*, *T*) are the field components along the two principal polarisation axes. The general propagation model for each fibre segment was based on the coupled generalised nonlinear Schrödinger equations (GNLSE) given by:

1 $$\begin{array}{ll}&({\partial }_{z}-{{{{{{{\rm{i}}}}}}}}{{\Delta }}{\beta }_{0}/2+{{\Delta }}{\beta }_{1}/2{\partial }_{T}+{{{{{{{\rm{i}}}}}}}}{\beta }_{2}/2{\partial }_{T}^{2}-{\beta }_{3}/6{\partial }_{T}^{3}-\hat{g}/2)u(z,T)=\\ &{{{{{{{\rm{i}}}}}}}}\gamma (1+{{{{{{{\rm{i}}}}}}}}/{\omega }_{0}{\partial }_{T})\left\{(1-{f}_{{{{{{{{\rm{R}}}}}}}}})\left[(| u{| }^{2}+2/3| v{| }^{2})u+1/3v^{2}{u}^{* }\right]+\right.\\ &\left.{f}_{{{{{{{{\rm{R}}}}}}}}}\ u(z,T){h}_{{{{{{{{\rm{R}}}}}}}}}(T)* (| u(z,T){| }^{2}+| v(z,T){| }^{2})\right\}\end{array}$$

2 $$\begin{array}{ll}&({\partial }_{z}+{{{{{{{\rm{i}}}}}}}}{{\Delta }}{\beta }_{0}/2-{{\Delta }}{\beta }_{1}/2{\partial }_{T}+{{{{{{{\rm{i}}}}}}}}{\beta }_{2}/2{\partial }_{T}^{2}-{\beta }_{3}/6{\partial }_{T}^{3}-\hat{g}/2)v(z,T)=\\ &{{{{{{{\rm{i}}}}}}}}\gamma (1+{{{{{{{\rm{i}}}}}}}}/{\omega }_{0}{\partial }_{T})\left\{(1-{f}_{{{{{{{{\rm{R}}}}}}}}})\left[(| v{| }^{2}+2/3| u{| }^{2})v+1/3{u}^{2}{v}^{* }\right]+\right.\\ &\left.{f}_{{{{{{{{\rm{R}}}}}}}}}\ v (z,T){h}_{{{{{{{{\rm{R}}}}}}}}}(T)* (| u(z,T){| }^{2}+| v(z,T){| }^{2})\right\}\end{array}$$

Here *β*_2_ and *β*_3_ are the second and third-order dispersion coefficients (assumed identical for each axis), and the weak (bend-induced) birefringence in each segment is included via the parameter Δ*β*_0_ = 2*π*/*L*_B_ where *L*_B_ is the beat length. A value of *L*_B_ = 5 m was used for all segments, consistent with the previous studies^31,79,80^. The group index term was calculated from the approximation Δ*β*_1_ ≈ Δ*β*_0_/*ω*_0_^81,82^. Jones calculus was used to model the effect of polarisation-selective elements such as waveplates and the polarising beamsplitter in the bulk cavity segment.

The usual Kerr nonlinear coefficient for each segment is *γ*, and higher-order nonlinear effects of self-steepening and Raman scattering were also included. The Raman contribution is included via the convolution (*) with the response function *h*_R_(*t*), which was based on a realistic model for silica^67,68^. Using a Raman fraction of *f*_R_ = 0.18 yielded good agreement with experiments, and we neglected orthogonal Raman gain contributions^83^. We stress that the use of this Raman model was essential to quantitatively reproduce the long-wavelength spectral broadening seen experimentally. We also note that although some previous studies have used a linear Raman approximation^84^, this cannot accurately describe the dynamics of sub-picosecond pulses^85^. We also stress here that the Raman fraction *f*_R_ is not a fitted parameter, but rather is determined from the peak of the Raman gain profile measured in experiment^86^. Although some small variation in the numerical value of the Raman fraction has been reported, the value of *f*_R_ = 0.18 has been found to yield good agreement with experiments as suggested in ref. ^68^.

Our modelling used fibre lengths and parameters based on the experimental cavity design. Segment AB consists of 11 m of OFS R37003 Erbium-doped fibre (EDF) with normal dispersion *β*_2_ = +40 × 10^−3^ ps^2^ m^−1^, and nonlinear parameter *γ* = 6.0 × 10^−3^ W^−1^ m^−1^. Third-order dispersion in the EDF was neglected. Standard silica fibre Segments BC, DE, and FA were of lengths 2.87 m, 4.45 m, and 7.8 m respectively, and used SMF-28 parameters *β*_2_ = −21.7 × 10^−3^ ps^2^ m^−1^, *β*_3_ = +86.0 × 10^−6^ ps^3^ m^−1^, and nonlinear parameter *γ* = 1.1 × 10^−3^ W^−1^ m^−1^. Although some cavity components (such as wavelength-selective couplers) used short lengths of other silica-based fibre, this was found to have a negligible effect on propagation and was not explicitly included in the modelling. The supercontinuum segment CD models propagation in 10.3 m of OFS highly nonlinear fibre with *β*_2_ = −5.23 × 10^−3^ ps^2^ m^−1^, *β*_3_ = +42.8 × 10^−6^ ps^3^ m^−1^ (zero-dispersion wavelength of 1408 nm), and nonlinear parameter *γ* = 18.4 × 10^−3^W^−1^ m^−1^. The net cavity dispersion is +0.06 ps^2^. Note that all dispersion and nonlinearity parameters above are specified at 1550 nm.

The gain term $\hat{g}(\omega )$ is non-zero only in the EDF segment, and we model this with a Lorentzian:

3 $$\hat{g}(\omega )=\frac{1}{1+E/{E}_{{{\mbox{sat}}}}}\times \frac{{g}_{0}}{1+{\Omega }^{2}/{\Omega }_{{{\mbox{g}}}\,}^{2}},$$

with *g*_0_ the unsaturated small-signal gain, *E* = ∫(∣*u*∣^2^ + ∣*v*∣^2^)*dτ* the intracavity pulse energy, and *E*_sat_ a gain saturation energy parameter. Ω = *ω* − *ω*_0_ is the detuned angular frequency, and *ω*_0_ is the central transition frequency (corresponding to a wavelength of 1550 nm) and Ω_g_ is the gain (half) bandwidth (corresponding to 20 nm). Note that this approach is widely used in the modelling of EDF amplifiers^13,87^ and is justified physically because gain recovery timescales for an Erbium-doped amplifier are typically ~100s of μs^88^, orders of magnitude slower than any of the characteristic timescales of our laser dynamics: the roundtrip time (179 ns); the noise-like pulse envelope (~100s of ps); the ultrafast soliton sub-structure (~100s of fs). We also note that because of the spectral filtering in the cavity, the bandwidth of the injected signal into the amplifier is effectively reset to ~10 nm for every roundtrip, which is significantly less than the 40 nm FWHM of the lineshape function. Moreover, there is no appreciable nonlinear spectral broadening in the EDF such that the pulses remain at ~10 nm bandwidth during amplification. This means that gain bandwidth-limiting effects^89^ are also negligible. Indeed, the fact that we can neglect nonlinear effects in the amplifier is another factor that allows us to focus more on the physics of the dramatic spectral broadening in the HNLF. We also note that our use of a constant distributed gain coefficient *g*_0_ is for consistency with previous studies of similar laser systems that have shown good agreement with experiments^13^. In fact, we explicitly checked that for our parameter regime, the average and statistical results from simulations do not significantly depend on the longitudinal gain model used. Finally, we note that typical polarisation-dependent gain in our parameter regime is expected to be below ~0.3 dB^90^, which can be neglected compared to our small signal and saturated gain of 35 and 21 dB, respectively, over the 11 m EDF length. Moreover, previous studies of polarisation-dependent gain saturation have also shown a negligible effect on nonlinear dynamics in dissipative soliton lasers^91^.

A bulk-optics free space segment EF (length 28.1 cm) includes a nonlinear-polarisation based saturable absorber^61,92^, and a narrowband spectral filter to control the bandwidth of the pulses reinjected into the EDF^62^. The filter transfer function was modelled on a double supergaussian fit to the experimentally measured intensity transmission function and was given by: $T(\Omega )={c}_{1}\exp (-{({{\Omega }}^{2}/{\Omega }_{1}^{2})}^{{m}_{1}})+{c}_{2}\exp (-{({{\Omega }}^{2}/{\Omega }_{2}^{2})}^{{m}_{2}})$ with coefficients *c*_1_ = 0.7036, *c*_2_ = 0.2944, *m*_1_ = 1.4483, *m*_2_ = 1.0034, Ω_1_ = 4.2584 × 10^12^ rad s^−1^, and Ω_2_ = 6.8006 × 10^12^ rad s^−1^. Linear losses in the cavity originate mainly from splicing and coupling and are considered at points C (0.84 dB), D (3.02 dB) and F (3.0 dB) in the simulation. The loss at point F includes the linear loss of the filter (20%).

Numerical simulations were performed using a 1.3 ns time window and 2^18^ = 262,144 points such that the temporal resolution is ~5 fs. The frequency grid corresponds to a wavelength span of ~1019–3238 nm with a frequency resolution of 769 MHz (wavelength resolution ~6 × 10^−3^ nm around 1550 nm). Although computationally very demanding, this level of time and frequency resolution is necessary to span the full noise-like pulse envelope structure, as well as to capture fine structure in the temporal and spectral domains. The numerical techniques used for the solution of GNLSE-like differential equations are well-known, and examples of numerical code for this purpose are widely available^63,68^.

A particular simulation is initiated at the input to the EDF (point A in Fig. 1a) using a gaussian noise background in the time-domain distributed across the full 1.3 ns time window^12,93^. The seed energy (distributed between the two polarisation components) was ~3 pJ. Physically, laser operation would be initiated by random amplified spontaneous emission noise from the EDF, but the averaged simulation results and the computed intensity peak statistics were found to be independent of the noise source used. Typically ~10^2^ roundtrips were required for the simulations to converge to the regime with well-defined mean energy, and it was only after entering this regime that statistical analysis was performed.

To compare simulations and experiments, we iteratively scanned the simulation parameter space to yield average spectral characteristics that agreed with the experiment (Fig. 7). This procedure yielded a small-signal gain of *g*_0_ = 0.73 m^−1^ and saturation energy of *E*_sat_ = 3.5 nJ. For the 225 mW pump power used in our experiments, these parameters were comparable to previous similar modelling of dissipative soliton lasers^93^, and supported by a rate equation analysis of the EDF single-pass gain characteristics^88^. The simulations yield a mean intracavity energy (averaged over 1000 roundtrips) of 10.5 nJ at the EDF output, compared with the experimental value of 13.6 nJ. Although the exact agreement would not be expected because of the approximate model of the gain lineshape function used, the overall agreement is remarkable between the mean energy, the average broadband spectrum with components spanning ~1000 nm, and computed temporal and spectral statistics. For completeness, we give the waveplate orientations for our simulations as (QWP1, HWP1, HWP2, QWP2) = (5. 4°, 16. 2°, 64. 8°, 27°) although a precise comparison with the experiment is not possible here because of the unknown birefringence orientation of each particular fibre segment in the cavity.

### Computation of the spectrogram

The simulations yield access to the amplitude and phase of the intracavity field, allowing us to calculate a frequency (or wavelength)-time spectrogram which clearly shows the intensity and phase content of the pulse in the time and frequency domains. We compute in particular the total field spectrogram *S*(*ω*, *τ*) = *S*_*u*_(*ω*, *τ*) + *S*_*v*_(*ω*, *τ*) where the separate component spectrograms are defined by:

4 $${S}_{k}(\omega ,\tau )={\left|\int\nolimits_{-\infty }^{\infty }g(T-\tau )f(T)\exp (-i\omega T){{{{{\mathrm{d}}}}}}T\,\right|}^{2}$$

with *k* = *u*, *v* and *f*(*T*) representing either *u*(*z*, *T*) or *v*(*z*, *T*) respectively, the field components along the two principal polarisation axes. The function *g*(*T* − *τ*), a variable delay gate function with delay value *τ*. The spectrogram trace then plots the spectra of a series of time-gated portions of the field and, especially when plotted against the associated temporal and spectral intensities, it provides a highly intuitive way to interpret the time-frequency structure of a complex field. In our calculation of the spectrogram, we used a gate function of 300 fs duration (full width at half maximum) and a Gaussian profile. Note that the use of the total spectrogram over both polarisations has the clear physical interpretation that it projects naturally onto the total temporal intensity profile and total spectrum.

Based on the spectrogram, it is possible to identify the wavelength of each of the Raman-shifted localised temporal peaks in the random HNLF output field, allowing calculation of the associated soliton number using ${N}_{{{{{{{{\rm{p}}}}}}}}}={[\gamma ({\omega }_{{{{{{{{\rm{p}}}}}}}}}){P}_{{{{{{{{\rm{p}}}}}}}}}{T}_{{{{{{{{\rm{p}}}}}}}}}^{2}/| {\beta }_{2}({\omega }_{{{{{{{{\rm{p}}}}}}}}})| ]}^{1/2}$. Here *γ*(*ω*_p_) and *β*_2_(*ω*_p_) are respectively the nonlinearity and dispersion parameters at the Raman-shifted peak frequency *ω*_p_, and *P*_p_ and *T*_p_ are respectively the pulse peak power and duration. The corrected dispersion parameter is then *β*_2_(*ω*_p_) = *β*_2_(*ω*_0_) + (*ω*_p_ − *ω*_0_) *β*_3_(*ω*_0_) where *ω*_0_ corresponds to a wavelength of 1550 nm. The corrected nonlinearity parameter is then *γ*(*ω*_p_) = (*ω*_p_/*ω*_0_)*γ*(*ω*_0_)^94^.

### Experimental setup

The laser system in Fig. 1 used a unidirectional cavity configuration with fibre lengths as described above. The overall laser repetition rate is 5.59 MHz (roundtrip time of ~179 ns). The primary laser output after the HNLF (point D) used a 40% coupler, and we used a 1% coupler at point C for diagnostics and a 5% coupler in segment DE for additional spectral measurement. The spectral filter used (Andover 155FSX-1025) had 10 nm bandwidth (FWHM) and 80% peak transmission. The EDF was co-directionally pumped at 976 nm, and noise-like pulsed behaviour was observed at all values of pump power above the 40 mW pump threshold where pulsed laser operation was first observed. In contrast to similar cavity configurations without the HNLF^28^, we did not observe any stable mode-locked regime for any combination of cavity parameters. With suitable adjustment of the waveplates, it was possible to observe noise-like pulse operation with broadband spectral output over a wide range of pump powers up to 500 mW. The observed spectra exhibited qualitatively similar characteristics over the full range of pump powers, although the broad temporal envelope duration (as measured with a photodiode) increased to ~450 ps at the highest pump powers. The results reported here at a pump power of 225 mW corresponded to 13 mW average power at the primary output after the HNLF (point D). This corresponds to intracavity energy of 7.3 nJ at the HNLF output and an energy at the EDF output of 13.6 nJ when accounting for all coupling and splicing losses between the EDF and HNLF.

Direct pulse envelope measurements used a 35 GHz photodiode (New Focus 1474 A) connected to a 20 GHz channel of a real-time oscilloscope (LeCroy 845 Zi-A, 40 GS s^−1^). The DFT setup used 5.13 km of dispersion compensating fibre (DCF) with group-velocity dispersion coefficient of *D* = −83.6 ps nm^−1^ km^−1^ (*β*_2_ = +107 × 10^−3^ ps^2^ m^−1^) at 1550 nm. The signal under test was attenuated before injection into the DCF to ensure linear propagation. The fidelity of the DFT measurements was confirmed by comparing the DFT spectrum with that measured using an averaging OSA (Anritsu MS9710B) for a separate stable mode-locked laser operating around 1550 nm. This comparison was also performed on the average spectra measured at the HNLF input (see results in Fig. 7b). The real-time DFT signal was measured by a 12.5 GHz photodiode (Miteq DR-125G-A) connected to another 20 GHz channel of the real-time oscilloscope, resulting in a spectral resolution of 0.19 nm. For the results in the inset of Fig. 7d, the broadband spectrum from the HNLF is plotted over an expanded wavelength range by combining measurements from the Anritsu MS9710B OSA below 1550 nm and the Ocean Optics NIRQuest512 spectrometer (resolution of 6.3 nm) above 1550 nm. The measured spectra were matched in the region of 1550 nm.

The time-lens setup was based on a commercial system (Picoluz UTM-1500) described in ref. ^95^, supplemented by an additional module of dispersion compensating fibre for additional magnification. A time-dependent quadratic phase was applied on the signal after propagation step *D*_1_ by four-wave mixing in a silicon waveguide between the signal and linearly-chirped pump pulses from an integrated fibre laser module (a 100 MHz Menlo C-Fiber Sync, a P100-EDFA and a pre-chirping fibre *D*_p_). The overall system magnification of ×190 was determined experimentally by using Fourier-domain pulse shaping (Finisar Waveshaper 4000 series) to create a picosecond pulse doublet with exactly 10 ps separation, and by measuring the increased temporal separation after passage through the time-lens. The magnification ∣*M*∣ = *D*_2_/*D*_1_ is associated with total dispersion for the input and output propagation steps of *D*_1_ = 4.16 ps nm^−1^ and *D*_2_ = 790.4 ps nm^−1^. The time-lens output was then recorded by a 12.5 GHz photodiode (Miteq DR-125G-A) connected to the 20 GHz channel of the real-time oscilloscope at a sampling rate of 40 Gs s^−1^. The calculated overall time-lens resolution^96^ was 340 fs, and the demagnified time window was ~100 ps (determined by the duration of the chirped pump pulses into the Silicon waveguide). Since the noise-like pulse envelope duration of ~300 ps is larger than the time-lens window, we operated in asynchronous mode with free-running acquisition triggered by the arrival of the time-lens signal. To avoid a low signal to noise ratio at the edges of the measurement window, all statistical analysis of the time-lens temporal peaks was performed only on the central (70 ps) region of the window.

### Rogue wave criteria

Based on the statistical distribution of intensity peaks in both the time and frequency domains, a significant intensity is defined as the associated mean intensity of the upper third of intensity peaks. The temporal and spectral rogue wave thresholds are defined as equal to 2.2 times this significant intensity^97^.

### Reporting summary

Further information on research design is available in the Nature Research Reporting Summary linked to this article.

## Supplementary information

Supplementary Information

Description of Additional Supplementary Files

Supplementary Movie 1

Reporting Summary

## Data Availability

Data are available from the corresponding author upon reasonable request.

## Code availability

The GNLSE simulation code used in this manuscript is available from the corresponding author upon reasonable request.
