# Supplementary material for: Intracavity incoherent supercontinuum dynamics and rogue waves in a broadband dissipative soliton laser
Source: Nat Commun. 2021 Sep 22;12:5567. doi: 10.1038/s41467-021-25861-4 (PMC8458443; doi:10.1038/s41467-021-25861-4)
Supplement: Supplementary file 1 — Supplementary Information [file 41467_2021_25861_MOESM1_ESM.pdf]

Supplementary Information: intracavity incoherent supercontinuum dynamics and rogue waves in a broadband dissipative soliton laser.

Fanchao Meng,[1] Coraline Lapre,[1] Cyril Billet,[1] Thibaut Sylvestre,[1] Jean-Marc Merolla,[1] Christophe Finot,[2] Sergei K. Turitsyn,[3,4] Goëry Genty,[5] and John M. Dudley[1,*]

[1] *Institut FEMTO-ST, Université Bourgogne Franche-Comté CNRS UMR 6174, 25000 Besançon, France*

[2] *Laboratoire Interdisciplinaire Carnot de Bourgogne, Université Bourgogne Franche-Comté CNRS UMR 6303, 21078 Dijon, France*

[3] *Aston Institute of Photonic Technologies, Aston University, Birmingham, UK*

[4] *Aston-NSU International Centre for Photonics, Novosibirsk State University, Novosibirsk, Russia.*

[5] *Photonics Laboratory, Tampere University, FI-33104 Tampere, Finland*

(Dated: August 31, 2021)

Abstract

We present additional numerical simulations of the dissipative soliton fibre laser described in the main manuscript, studying in particular how the length of highly nonlinear fibre in the cavity influences the laser dynamics.

[*] Corresponding Author:john.dudley@univ-fcomte.fr

SUPPLEMENTARY DISCUSSION

We present here additional simulation results varying the length of highly nonlinear fibre (HNLF) for a range of intracavity pulse energies. Our results below (and the textual labels) refer to the laser cavity shown in Fig. 1 of the main manuscript. Simulations consider HNLF lengths of $|CD| = L_{\mathrm{HNLF}} = 0$ m, 1.3 m, 5.3 m, and 10.3 m. The value of 10.3 m are those also shown in the main manuscript, and corresponds to the length used in experiment. These results allow us to better understand the transition from stable to unstable operation, and to see more explicitly the dynamical and operational differences observed between the narrowband and broadband noise-like pulse regimes. All other laser parameters and the simulation methodology are as described in the Methods section of the main manuscript.

We first present simulation results without any HNLF in the cavity (i.e. $L_{\mathrm{HNLF}} = 0$ m). We show results for different values of the saturation parameter E_{sat}, allowing us to see how the laser dynamics change with intracavity energy [1]. These results are shown in Supplementary Fig. 1 for saturation energies of: (a) $E_{\mathrm{sat}} = 0.18$ nJ, (b) $E_{\mathrm{sat}} = 2$ nJ, (c) $E_{\mathrm{sat}} = 3.5$ nJ, corresponding to intracavity energies (at the EDF output) of: (a) $E\mathrm{cav} = 0.6$ nJ, (b) $E\mathrm{cav} = 6.9$ nJ, (c) $E\mathrm{cav} = 13$ nJ. For each case we show: (i) false colour maps of the spectral (left) and temporal (right) evolution over one roundtrip and (ii) the temporal and spectral profiles at the output of the SMF (point C), the point that would usually be the segment between the EDF and HNLF.

The results in Supplementary Figs. 1(a) and 1(b) at lower energies do not show noise-like pulse behaviour but rather show convergence to: (a) a stable single pulse state, and (b) a stable phase-locked soliton molecule. The evolution shows the breathing of the intracavity spectral and temporal profiles as the pulse encounters fibre elements with different signs of dispersion, which are similar for both the single pulse and molecule states. A difference in the case of the molecule state is of course the presence of spectral fringes which confirms the soliton phase-locking in this regime [2]. The transition from single-soliton to soliton molecules for increased saturation energy is expected, and are similar to many previous numerical and experimental studies [3–5].

The results in Supplementary Fig. 1(c) at higher energy shows the transition to the unstable noise-like pulse regime. Both the spectral and temporal evolution are incoherent, exhibiting typical features of fibre laser instabilities such as soliton spectral explosions [4, 6]. The (unstable) spectral bandwidth (FWHM) in the cavity remains limited to a maximum of around ∼40 nm, such that these results correspond to the narrowband regime of noise-like pulse operation where modulation-instability-like incoherent soliton evolution is at the origin of the shot-to-shot fluctuations [7–14].

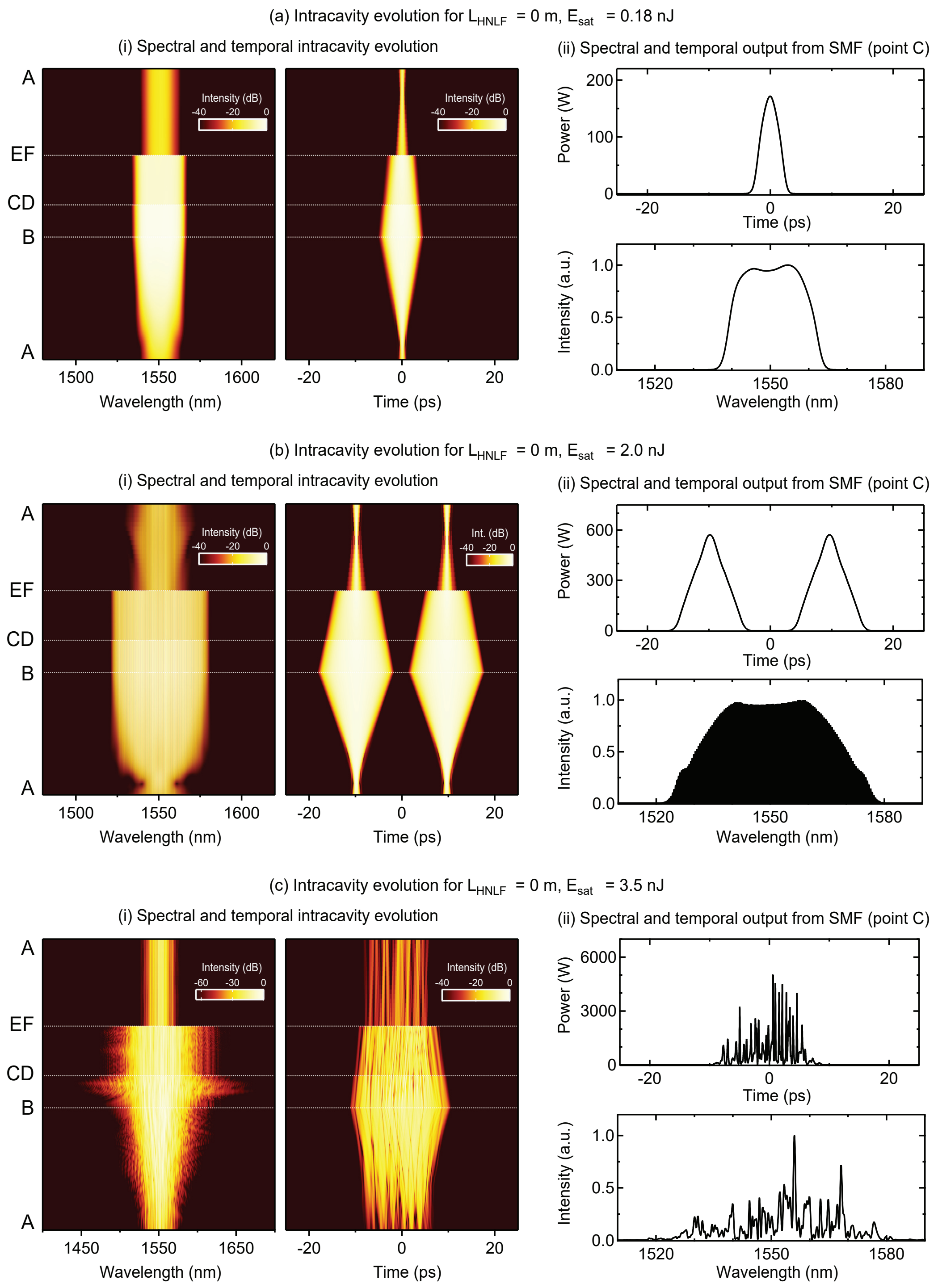

Supplementary Figure 1. Simulation results for $L_{\mathrm{HNLF}} = 0$ m and saturation energies of (a) $E_{\mathrm{sat}} = 0.18$ nJ, (b) $E_{\mathrm{sat}} = 2$ nJ, (c) $E_{\mathrm{sat}} = 3.5$ nJ. For each case we show: (i) Intracavity spectral (left) and temporal (right) evolution over one roundtrip; (ii) Temporal (top) and spectral (bottom) profiles at the SMF output (point C). Labels A–F refer to Fig. 1 in the main manuscript.

Supplementary Figs 2 and 3 present simulation results with the addition of HNLF. Supplementary Fig. 2 shows results for only a short length of $L_{\text{HNLF}} = 1.3$ m, at saturation energies of: (a) $E_{\text{sat}} = 0.18$ nJ, (b) $E_{\text{sat}} = 2$ nJ, (c) $E_{\text{sat}} = 3.5$ nJ. This corresponds to intracavity energies (at the EDF output) of: (a) $E\text{cav} = 0.6$ nJ, (b) $E\text{cav} = 5.7$ nJ, (c) $E\text{cav} = 9.9$ nJ. For each case we show: (i) Intracavity spectral (left) and temporal (right) evolution over one roundtrip; (ii) Temporal (top) and spectral (bottom) profiles at the HNLF output (point D).

At the lowest saturation energy of 0.18 nJ, the results in Supplementary Fig. 2(a) are qualitatively similar to the results in Supplementary Fig. 1(a) in that we see stable single pulse evolution. However, the higher values of saturation energy in Supplementary Figs 2(b) and 2(c) give dramatically different noise-like pulse dynamics. Specifically, we see how even a small length of HNLF leads to significant spectral broadening (spanning over 500 nm) and, although we cannot clearly see distinct Raman soliton evolution in the spectrum, we can infer the presence of soliton dynamics in the HNLF through the clear dispersive wave component apparent around 1150 nm. We also see signatures of soliton localisation in the time-domain evolution map, and in the single shot temporal profile in subfigure (ii).

To show the effect of HNLF length on the dynamics more explicitly, Supplementary Fig. 3 presents additional simulation results for a fixed saturation energy of 3.5 nJ and three different HNLF lengths of (a) 1.3 m, (b) 5.3 m, and (c) 10.3 m. The corresponding intracavity energies (at the EDF output) are: (a) 9.9 nJ, (b) 9.9 nJ, and (c) 10.5 nJ. These results were all associated with irregular noise-like pulse characteristics, and so all evolution plots are typical single-shot results over one roundtrip. The corresponding average spectra over 1000 roundtrips were also calculated, and are shown in plots (d),(e),(f).

The spectral evolution plots in Supplementary Figs. 3(a)-(c) are significant in showing the enhancement in spectral extension that results from the increased Raman soliton self-frequency shift at greater lengths of HNLF. This trend is also manifested in the average spectra (at the HNLF output) computed over 1000 roundtrips shown in Supplementary Figs. 3(d)-(f). It is clear how the Raman dynamics in the HNLF play the critical role in extending the spectra to longer wavelengths.

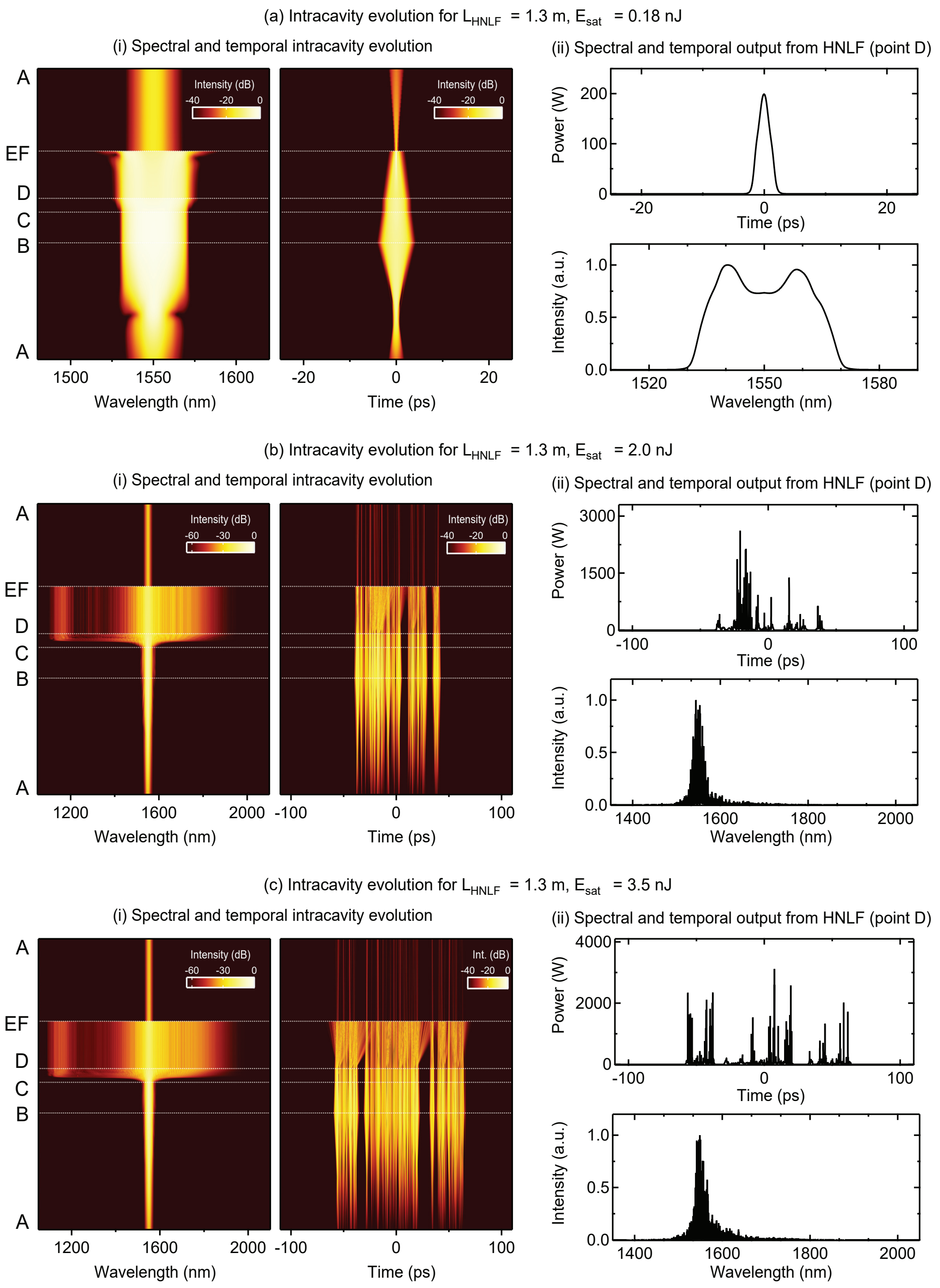

Supplementary Figure 2. Simulation results for $L_{\mathrm{HNLF}} = 1.3$ m and saturation energies of (a) $E_{\mathrm{sat}} = 0.18$ nJ, (b) $E_{\mathrm{sat}} = 2$ nJ, (c) $E_{\mathrm{sat}} = 3.5$ nJ. For each case we show: (i) Intracavity spectral (left) and temporal (right) evolution over one roundtrip; (ii) Temporal (top) and spectral (bottom) profiles at the HNLF output (point D).

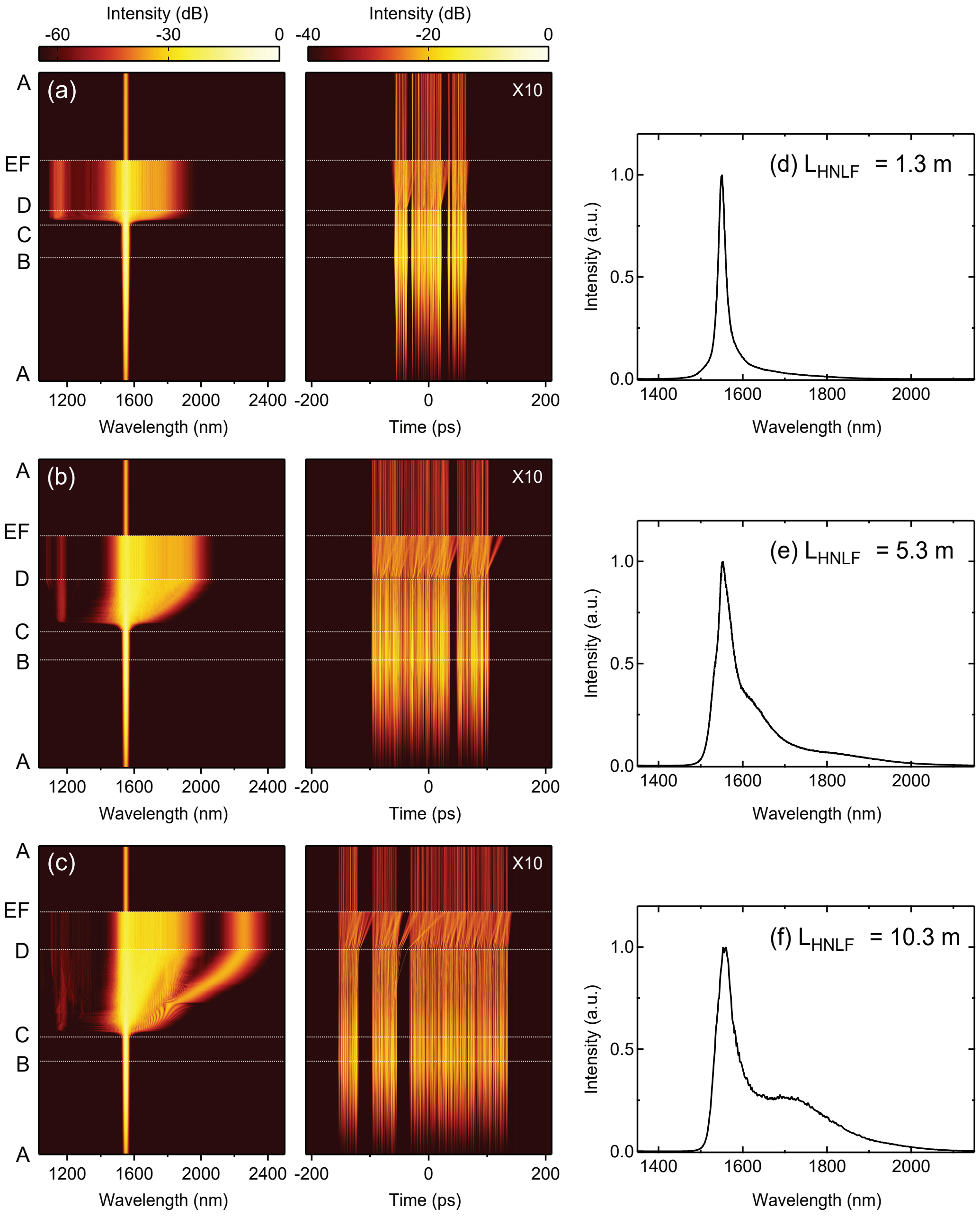

Supplementary Figure 3. Simulated spectral and temporal evolution for a fixed saturation energy of 3.5 nJ and HNLF lengths of (a) 1.3 m, (b) 5.3 m, and (c) 10.3 m. All evolution plots are typical single-shot results over one roundtrip. The corresponding average spectra (at the HNLF output) computed over 1000 roundtrips are shown in the corresponding plots (d),(e),(f).

SUPPLEMENTARY REFERENCES

[1] Milonni, P. W. & Eberly, J. H. *Laser Physics* (John Wiley & Sons, 2010).

[2] Herink, G., Kurtz, F., Jalali, B., Solli, D. R. & Ropers, C. Real-time spectral interferometry probes the internal dynamics of femtosecond soliton molecules. *Science* **356**, 50–54 (2017).

[3] Woodward, R. I. Dispersion engineering of mode-locked fibre lasers. *Journal of Optics* **20**, 033002 (2018).

[4] Lapre, C. *et al.* Real-time characterization of spectral instabilities in a mode-locked fibre laser exhibiting soliton-similariton dynamics. *Scientific Reports* **9**, 13950 (2019).

[5] Meng, F., Lapre, C., Billet, C., Genty, G. & Dudley, J. M. Instabilities in a dissipative soliton-similariton laser using a scalar iterative map. *Optics Letters* **45**, 1232–1235 (2020).

[6] Runge, A. F. J., Aguergaray, C., Broderick, N. G. R. & Erkintalo, M. Coherence and shot-to-shot spectral fluctuations in noise-like ultrafast fiber lasers. *Optics Letters* **38**, 4327 (2013).

[7] Horowitz, M., Barad, Y. & Silberberg, Y. Noiselike pulses with a broadband spectrum generated from an erbium-doped fiber laser. *Opt. Lett.* **22**, 799–801 (1997).

[8] Tang, D. Y., Zhao, L. M. & Zhao, B. Soliton collapse and bunched noise-like pulse generation in a passively mode-locked fiber ring laser. *Opt. Express* **13**, 2289–2294 (2005).

[9] Kobtsev, S., Kukarin, S., Smirnov, S., Turitsyn, S. & Latkin, A. Generation of double-scale femto/pico-second optical lumps in mode-locked fiber lasers. *Opt. Express* **17**, 20707–20713 (2009).

[10] Pottiez, O., Grajales-Coutiño, R., Ibarra-Escamilla, B., Kuzin, E. & Hernández-García, J. Adjustable noiselike pulses from a figure-eight fiber laser. *Applied Optics* **50**, E24–E31 (2011).

[11] Lecaplain, C. & Grelu, P. Rogue waves among noiselike-pulse laser emission: An experimental investigation. *Physical Review A* **90**, 013805 (2014).

[12] Li, B. *et al.* Unveiling femtosecond rogue-wave structures in noise-like pulses by a stable and synchronized time magnifier. *Optics Letters* **44**, 4351–4354 (2019).

[13] Wang, Z., Nithyanandan, K., Coillet, A., Tchofo-Dinda, P. & Grelu, P. Buildup of incoherent dissipative solitons in ultrafast fiber lasers. *Physical Review Research* **2**, 013101 (2020).

[14] Du, Y. *et al.* Alternation of the mode synchronization and desynchronization in ultrafast fiber laser. *Laser & Photonics Reviews* **14**, 1900219 (2020).
