# Supplementary material for: Intracavity incoherent supercontinuum dynamics and rogue waves in a broadband dissipative soliton laser
Source: Nat Commun. 2021 Sep 22;12:5567. doi: 10.1038/s41467-021-25861-4 (PMC8458443; doi:10.1038/s41467-021-25861-4)
Supplement: Supplementary file 2 — Description of Additional Supplementary Files [file 41467_2021_25861_MOESM2_ESM.pdf]

Description of Additional Supplementary Files

Supplementary Movie 1. Evolution of the intracavity field over one roundtrip in the laser cavity. The figure shows the computed spectrogram together with the corresponding projected temporal and spectral intensity profiles. At each point in the evolution, the position in the cavity (measured from the input to the Erbium doped amplifier) in the cavity schematic is also shown.
