# Supplementary material for: Intracavity incoherent supercontinuum dynamics and rogue waves in a broadband dissipative soliton laser
Source: Nat Commun. 2021 Sep 22;12:5567. doi: 10.1038/s41467-021-25861-4 (PMC8458443; doi:10.1038/s41467-021-25861-4)
Supplement: Supplementary file 4 — Reporting Summary [file 41467_2021_25861_MOESM4_ESM.pdf]

nature research

Corresponding author(s): John Dudley (john.dudley@univ-fcomte.fr)

Lasing Reporting Summary

Nature Research wishes to improve the reproducibility of the work that we publish. This form is intended for publication with all accepted papers reporting claims of lasing and provides structure for consistency and transparency in reporting. Some list items might not apply to an individual manuscript, but all fields must be completed for clarity.

For further information on Nature Research policies, including our data availability policy, see Authors & Referees.

~ Experimental design

Please check: are the following details reported in the manuscript?

1. Threshold

Plots of device output power versus pump power over a wide range of values indicating a clear threshold

- [ ] Yes
- [x] No

Since our work describes the noise-like pulse unstable regime of an existing laser operating far above the threshold rather than a new report of lasing per se, we did not feel it necessary to provide the typical "laser graph" showing output power vs pump from well below threshold. Rather, we focused on providing the most relevant parameter for this study which is the laser mode-locking threshold value of 40 mW. This said, we have of course measured the full output power vs pump laser curve and can provide this on request.

2. Linewidth narrowing

Plots of spectral power density for the emission at pump powers below, around, and above the lasing threshold, indicating a clear linewidth narrowing at threshold

- [ ] Yes
- [x] No

We provide many plots of the laser spectral characteristics above the mode-locking threshold to illustrate the complex noise-like pulse dynamics which is the main focus of the paper. We have of course fully characterized the standard fiber laser behavior at lower pump powers at the onset of continuous wave lasing (threshold ~5-10 mW depending on waveplate orientation) where we see the usual changes in the spectrum from ASE to narrowband. Although these data are not directly related to our study which focusses on the more complex random pulse dynamics in the noise-like pulse regime, we can provide on request.

Resolution of the spectrometer used to make spectral measurements

- [x] Yes
- [ ] No

Yes, our Methods section gives the spectral resolution.

3. Coherent emission

Measurements of the coherence and/or polarization of the emission

- [x] Yes
- [ ] No

Studying the stability properties of this noise-like source is a particular aim of our paper, and we report measurements in both the time and frequency domain that use real-time time-lens and dispersive Fourier Transform characterization to directly measure shot-to-shot variations in the laser properties. From these measurements, we would be able to extract corresponding intensity correlations (second order coherence) in the time and spectral domain. This said, our direct measurements of the temporal and spectral fluctuations are actually of more relevance to determine statistical distributions and to identify rogue wave events. Measurements of first order coherence are not directly relevant to our study of the noise-like pulse regime.

4. Beam spatial profile

Image and/or measurement of the spatial shape and profile of the emission, showing a well-defined beam above threshold

- [ ] Yes
- [x] No

Being a fiber-laser, the emission characteristics necessarily reflect the modal guidance properties of the fiber used which are single mode above ~1250 nm. The output after collimation is a well-defined single mode beam, and we did not see any evidence of multimode behavior. We give the fiber types in the main text so this should be apparent to a reader.

5. Operating conditions

Description of the laser and pumping conditions
Continuous-wave, pulsed, temperature of operation

- [x] Yes
- [ ] No

Yes, a full experimental description is given together with a schematic, and all component part numbers, fiber lengths, and pump powers.

Threshold values provided as density values (e.g. W cm^{-2} or J cm^{-2}) taking into account the area of the device

- [ ] Yes
- [x] No

We did not specify the threshold as a density value as this is not usual practice for fiber lasers. However we do give the threshold power in W and the part number (OFS R37003) of the Er-doped fiber used in experiment. It is straightforward for any threshold density to be calculated if required.

6. Alternative explanations

Reasoning as to why alternative explanations have been ruled out as responsible for the emission characteristics
e.g. amplified spontaneous, directional scattering; modification of fluorescence spectrum by the cavity

☐ Yes
☒ No

Since we are building on an established ring cavity design for fiber lasers, this is not a necessary consideration.

7. Theoretical analysis

Theoretical analysis that ensures that the experimental values measured are realistic and reasonable
e.g. laser threshold, linewidth, cavity gain-loss, efficiency

☒ Yes
☐ No

Yes, we fully report a theoretical and numerical model of our laser, and indeed this is one of the major novelties in that we are able to show quantitative agreement between simulation and experiment with supercontinuum emission over a broad wavelength range.

8. Statistics

Number of devices fabricated and tested

☐ Yes
☒ No

Since we are building on an established ring cavity design for fiber lasers, this is not a necessary consideration.

Statistical analysis of the device performance and lifetime (time to failure)

☐ Yes
☒ No

Since we are building on an established ring cavity design for fiber lasers that are known to be highly robust, we did not consider it necessary to include a discussion of long term performance. However, the laser has been operating near-continuously in our laboratories for 12 months and as with any fiber laser, the failure time is limited by the pump source, but this is typically 10,000 hours or more.
